# Microbial Community Structure of Subglacial Lake Whillans, West Antarctica

**DOI:** 10.3389/fmicb.2016.01457

**Published:** 2016-09-22

**Authors:** Amanda M. Achberger, Brent C. Christner, Alexander B. Michaud, John C. Priscu, Mark L. Skidmore, Trista J. Vick-Majors, the WISSARD Science Team, W. Adkins

**Affiliations:** ^1^Department of Biological Sciences, Louisiana State University, Baton RougeLA, USA; ^2^Department of Microbiology and Cell Science, University of Florida, GainesvilleFL, USA; ^3^Biodiversity Institute, University of Florida, GainesvilleFL, USA; ^4^Department of Land Resources and Environmental Science, Montana State University, BozemanMT, USA; ^5^Department of Earth Sciences, Montana State University, BozemanMT, USA

**Keywords:** Antarctica, subglacial lake, subsurface microbiology, biogeochemical cycling, chemosynthetic ecosystem

## Abstract

Subglacial Lake Whillans (SLW) is located beneath ∼800 m of ice on the Whillans Ice Stream in West Antarctica and was sampled in January of 2013, providing the first opportunity to directly examine water and sediments from an Antarctic subglacial lake. To minimize the introduction of surface contaminants to SLW during its exploration, an access borehole was created using a microbiologically clean hot water drill designed to reduce the number and viability of microorganisms in the drilling water. Analysis of 16S rRNA genes (rDNA) amplified from samples of the drilling and borehole water allowed an evaluation of the efficacy of this approach and enabled a confident assessment of the SLW ecosystem inhabitants. Based on an analysis of 16S rDNA and rRNA (i.e., reverse-transcribed rRNA molecules) data, the SLW community was found to be bacterially dominated and compositionally distinct from the assemblages identified in the drill system. The abundance of bacteria (e.g., *Candidatus* Nitrotoga, *Sideroxydans, Thiobacillus*, and *Albidiferax*) and archaea (*Candidatus* Nitrosoarchaeum) related to chemolithoautotrophs was consistent with the oxidation of reduced iron, sulfur, and nitrogen compounds having important roles as pathways for primary production in this permanently dark ecosystem. Further, the prevalence of *Methylobacter* in surficial lake sediments combined with the detection of methanogenic taxa in the deepest sediment horizons analyzed (34–36 cm) supported the hypothesis that methane cycling occurs beneath the West Antarctic Ice Sheet. Large ratios of rRNA to rDNA were observed for several operational taxonomic units abundant in the water column and sediments (e.g., *Albidiferax, Methylobacter, Candidatus* Nitrotoga, *Sideroxydans*, and *Smithella*), suggesting a potentially active role for these taxa in the SLW ecosystem. Our findings are consistent with chemosynthetic microorganisms serving as the ecological foundation in this dark subsurface environment, providing new organic matter that sustains a microbial ecosystem beneath the West Antarctic Ice Sheet.

## Introduction

Remote sensing and field surveys have identified ∼400 lakes beneath the Antarctic ice sheet (Smith et al., 2009; Wright and Siegert, 2012), provided evidence for the widespread occurrence of water-saturated sediments (e.g., Studinger et al., 2001), and demonstrated hydrologic connections between certain lakes (Fricker et al., 2007). The abundance of water and availability of metabolic energy sources beneath the ice sheet (Bell, 2008; Smith et al., 2009; Skidmore, 2011; Palmer et al., 2013) supports the possibility for microbial ecosystems and has motivated international exploration efforts in West and East Antarctica (Fricker et al., 2011; Lukin and Bulat, 2011; Ross et al., 2011). Although little information has been available to infer conditions in Antarctica’s vast and complex subglacial aquifer, microbial cells inhabiting this biome are estimated at ∼10^29^ (Priscu et al., 2008), a value similar to global estimates for open ocean waters (Whitman et al., 1998). Fortunately, recent progress in subglacial exploration (Tulaczyk et al., 2014) has provided an opportunity to directly examine the nature, evolution, and biogeochemical contributions of microbial communities at the bed of Antarctica’s ice sheet.

Much of the data amassed on subglacial microbial communities has been derived through analyses of subglacial water outflows from alpine and polar glaciers (Skidmore et al., 2005; Cheng and Foght, 2007; Mikucki and Priscu, 2007; Boyd et al., 2011; Hamilton et al., 2013; Dieser et al., 2014) or basal materials retrieved from deep ice boreholes (e.g., Priscu et al., 1999; Lanoil et al., 2009; Christner et al., 2012). This approach has been valuable for providing baseline data and identifying common trends in disparate subglacial ecosystems. For example, there is geochemical and microbiological evidence that subglacial microbes derive their energy through the weathering of bedrock minerals and thus influence subglacial water chemistry (Skidmore, 2011; Mitchell et al., 2013). The prevalence of phylotypes related to species of *Thiobacillus* and *Sideroxydans* suggests primary production at the glacier bed may rely on reduced iron and sulfur compounds liberated through glacial comminution and microbiological processes occurring in the sediments or at the bedrock interface (Skidmore et al., 2010; Boyd et al., 2014). Evidence for the activity of methanogenic, methanotrophic, and ammonia oxidizing species has also been provided in several subglacial environments (Boyd et al., 2010, 2011; Dieser et al., 2014), implying these pathways could also play important roles in carbon and nitrogen cycling beneath larger ice masses. Although these pioneering efforts have provided valuable data to generate hypotheses on the structure and function of subglacial microbial ecosystems, their applicability to environments beneath ice sheets has remained uncertain.

Directly sampling sub-ice aquatic environments in a microbiologically clean manner is logistically challenging (Doran et al., 2008; Siegert et al., 2012), requiring strategies to reduce microbial cells associated with the drilling process and minimize exchange between the surface and subglacial environment (Priscu et al., 2013). During January 2013, the Whillans Ice Stream Subglacial Access Research Drilling (WISSARD) Project conducted the first successful sampling of an Antarctic subglacial lake (Christner et al., 2014; Tulaczyk et al., 2014). Christner et al. (2014) reported that planktonic bacteria and archaea in the aerobic water column were at an average concentration of 1.3 × 10^5^ cells mL^-1^ and morphologically diverse. Molecular analysis of 16S rRNA gene sequences amplified from the water column and surficial sediments (0–2 cm) revealed a rich prokaryotic community consisting of several phylotypes similar to chemosynthetic species that have been observed in alpine and polar glacier environments (e.g., members of *Thiobacillus, Sideroxydans*, and *Methylobacter* (Lanoil et al., 2009; Boyd et al., 2014; Dieser et al., 2014). Furthermore, primary and heterotrophic production data revealed that Subglacial Lake Whillans (SLW) contained a metabolically functional microbial community that may be sustained by dark autotrophic activity (Christner et al., 2014).

Here we present a detailed description of SLW’s water column and sediment (to depths of 36 cm) communities based on analysis of amplified 16S rRNA genes (rDNA) and molecules (rRNA). This approach served the dual role of providing information on microbial community structures while also allowing an assessment of potentially metabolically active taxa and the biogeochemical reactions they are likely to catalyze. Our data provide an initial framework for discerning the diversity and ecology of Antarctic subglacial lake environments, and support the hypotheses that microbial transformations beneath ice masses are driven by chemosynthesis and have global biogeochemical significance (Wadham et al., 2012).

## Materials and Methods

### Site Description and Drilling Operations

SLW is centrally located in the lower Whillans Ice Stream (WIS), West Antarctica beneath ∼800 m of ice and has a maximum area of ∼60 km^2^. The water column depth was ∼2.2 m when sampled in January 2013 (Fricker and Scambos, 2009; Christianson et al., 2012; Tulaczyk et al., 2014). Observations of ice surface elevation changes in this region of the WIS have provided data to infer subglacial hydrological conditions and examine their influence on ice sheet behavior (Bell, 2008; Pritchard et al., 2012; Carter et al., 2013). SLW was shown to receive episodic water input from the upper WIS and the neighboring Kamb Ice Stream, and as such, is classified as an active lake (Smith et al., 2009; Wright and Siegert, 2012). Since 2003, SLW has filled and drained three times (Siegfried et al., 2016). The outflow is transported via subglacial channels ∼100 km to the grounding zone and drains into the marine cavity beneath the Ross Ice Shelf (Fricker and Scambos, 2009). At the time of sampling, SLW was at a low stand and filling (Siegfried et al., 2016). The surface sediments of the lake bed were composed primarily of glacial till that was likely deposited during flood events (Hodson et al., 2016), while the deeper sediments contained seawater signatures that linked their origin to past marine intrusions in the region (Christner et al., 2014; Michaud et al., 2016).

During January 2013, a hot water drilling system (Blythe et al., 2014; Burnett et al., 2014; Rack et al., 2014) was used to create a ∼0.6 m diameter borehole (location 84.240°S, 153.694°W) in the ice overlying SLW. The borehole provided direct access to the SLW water column and underlying sediments over a period of 3 days. Details on the scientific operations conducted by WISSARD at SLW are provided in Tulaczyk et al. (2014).

### Microbiological Monitoring during Hot Water Drilling

To minimize the introduction of microbial contamination into SLW during scientific operations, the melted snow and ice used for drilling was sequentially passed through large capacity filters (2.0 and 0.2 μm) and irradiated with UV (185 and 245 nm) to remove and kill cells, respectively. Subsequently, the water was heated to ∼90°C and pumped through ∼1 km of drill hose into the borehole (Supplementary Figure S1A). The WISSARD water treatment system was tested extensively and its effectiveness is described in Priscu et al. (2013).

Water was sampled from various ports in the hot water system during drilling and in the borehole via hydrocast (Supplementary Figure S1A) to assess the cell concentration and microbial diversity of the drill water. Once drilling progressed to a depth of ∼210 m below the surface (mbs), 19 L of water was collected from an access port located before the filtration and UV systems and designated sample T1P1 (i.e., time point 1, port 1). Simultaneously, an equal volume from the return pump (designated T1P9) that recirculated water from the borehole to the water treatment system was sampled (Supplementary Figure S1A; Rack, 2016). Drilling was paused at a depth of ∼700 mbs to allow visual inspection of the borehole and water sampling. A 10 L Niskin bottle was deployed to retrieve a direct sample of the borehole water from a depth of 672 mbs (Supplementary Figure S1A). After drilling resumed and the borehole reached a depth of ∼735 mbs, water samples were again collected from ports on the drilling system. Water returning from the borehole was designated T2P9 (time point 2, port 9) and that originating from a port located after the water heater were designated T2P8 (Supplementary Figure S1A). Immediately after collection, cells in the water samples were filter concentrated on 142 mm, 0.2 μm Supor membrane filters (Pall Corp.) using a peristaltic pump and sterile tubing.

To disinfect the equipment and instrumentation used during borehole operations, 3% hydrogen peroxide (w/v) was applied to all surfaces with a pressure sprayer and the cleaned materials were staged in sealed polyethylene bags. The filter housing of the Large Volume Water Transfer System (WTS-LV; McLane Research Laboratories, Inc.) was cleaned between casts by sequential washes with sterile water, 70% ethanol, and 3% hydrogen peroxide.

### Water Column and Sediment Sampling

Particulates in the SLW water column were concentrated by filtration *in situ* using a WTS-LV that was modified for borehole deployment. During 68 h of borehole operations, the WTS-LV was deployed three times (Tulaczyk et al., 2014). Each cast was made at the approximate middle of the 2.2 m water column and filtration occurred for ∼2 h. Particulates in the water were collected using a custom, modular 142 mm polyvinyl chloride (PVC) filter holder and sequentially concentrated on Supor membrane filters (Pall Corp.) with pore sizes of 10, 3, 0.8, and 0.2 μm. Flow was measured with an analog meter on the WTS-LV, and 4.9, 5.3, and 7.2 L of water was concentrated during the first, second, and third casts, respectively. Immediately upon retrieval from the borehole, the filter housing was transferred to a class 100 laminar flow hood (Labconco) in the field laboratory. The filters were removed from the housing, placed in sterile 142 mm Petri dishes, and quartered with a sterile scalpel. Each quarter was placed in a 7 mL cryovial and stabilized by the addition of 5 mL of RNAlater (Ambion) or DNA buffer solution [40 mM ethylenediaminetetraacetic acid (EDTA) pH 8.0, 50 mM Tris pH 8.3, 0.73 M sucrose].

A 36 cm long sediment core was collected using a multicoring device (Uwitec). The core was sectioned at 2 cm intervals in a class 100 laminar flow hood (Labconco), and 7–15 g from the inner portion of each interval was placed in sterile 60 mL Nalgene bottles, preserved in either RNAlater or DNA buffer solution, and mixed. Filters and sediments amended with RNAlater were incubated at 4°C for 10–12 h before freezing. All samples were stored at -80°C until analyzed.

### Nucleic Acid Extraction, Amplification, and Sequencing

DNA was extracted from filters and sediment samples using the Power Water and Power Soil DNA Isolation kits (MO BIO Laboratories, Inc.), respectively, according to the manufacturer’s protocols. Amplification and sequencing of 16S rRNA genes was conducted as previously described (Christner et al., 2014). RNA was extracted from cells on the filters and reverse transcribed into complementary DNA (cDNA) using the method described by Dieser et al. (2014). For sediment samples, 3 g (wet weight) were centrifuged at 25,000 × *g* for 10 min. The supernatant was discarded and the sediment pellet was suspended in 3 mL of Tris EDTA (TE) buffer (1 mM EDTA, 10 mM Tris; pH 6.3). The slurry was subsequently processed with the same extraction protocol used for the filters. Procedural blanks that consisted of clean filters were extracted in parallel and served as methodological controls.

Amplification of the V4 region of the 16S rRNA gene was performed in a 50 μL volume using 5 units of AmpliTaq Gold DNA Polymerase LD (Invitrogen) and the following components; 1× PCR Gold Buffer, 3.5 mM MgCl_2_, 10 pmol of each primer (515F and 806R; Caporaso et al., 2012), 200 μM deoxynucleotides (dNTPs) and 1.5–5 μL of DNA template (0.02–1 ng). The polymerase chain reaction (PCR) consisted of a 5–8 min initial denaturation at 94°C followed by 35 cycles of amplification under the following conditions: 94°C for 45 s, 50°C annealing for 90 s, and elongation at 72°C for 90 s, with a terminal elongation at 72°C for 10 min. Methodological controls and some of the sediment samples were amplified using up to 40 cycles when no or weak amplification was observed after 35 cycles of amplification. PCR products were evaluated using agarose gel electrophoresis and amplicon concentrations were determined using the Quant-iT PicoGreen dsDNA Assay Kit (Life Technologies). Several sediment extractions showed poor amplification and were not submitted for sequencing. The amplicons were pooled in equimolar concentrations, and primers and PCR reagents were removed with the MoBio UltraClean PCR Clean-Up Kit. The pooled amplicons were sequenced on an Illumina MiSeq platform that generated 250 bp paired end reads.

### Phylogenetic and Statistical Analysis

Paired end sequence reads from the V4 region of the 16S rRNA gene (Caporaso et al., 2012) were assembled into contigs, quality filtered using the mothur phylogenetic analysis pipeline (v1.33.3; Schloss et al., 2009), and aligned with the mothur-compatible version of the SILVA database (v119). Chimeric sequences were identified and removed using the Uchime algorithm (Edgar et al., 2011), as implemented within mothur. All sequences with ≥97% 16S rRNA gene sequence similarity were defined as an operational taxonomic unit (OTU), and a representative sequence for each OTU was classified using the SEED database with the SILVA Incremental Aligner (SINA v1.2.11; Pruesse et al., 2012). Diversity and richness estimations (Shannon, Inverse Simpson, and Chao1) were calculated using mothur on OTU abundance data normalized to the smallest sample dataset (25,904 sequences; Supplementary Table S1). Singletons were removed before further analysis. Abundant OTUs were defined as having a relative abundance ≥1% of the sequences from a given sample. OTUs that were abundant in the drilling and methodological control samples were designated as potential contaminants and not considered as members of the SLW community. Non-metric multidimensional scaling (NMDS) plots were computed within R (v.3.1.2; R Core Team, 2015) on normalized data that was logarithmically transformed prior to calculation of Bray Curtis dissimilarity matrices using the Vegan (decostand and vegdist functions; Oksanen et al., 2013) and labdsv (nmds function; Roberts, 2007) packages. The level of similarity between microbial communities of various sample groups was further evaluated using analysis of molecular variance, implemented within mothur. Sequence data are available in the NCBI Sequence Read Archive under project PRJNA244335.

## Results

### Composition of the Abundant Bacterial and Archaeal OTUs in SLW

**FIGURE 1:**
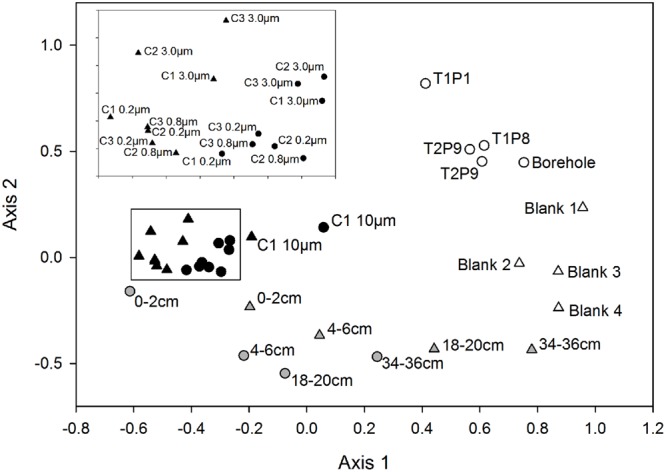
**Non-metric multidimensional scaling plot based on OTUs classified from the 16S rRNA and rRNA gene sequences obtained from samples of the drilling water, SLW water column, sediments, and experimental controls.** Circles represent rDNA-based sample libraries and triangles represent rRNA-based libraries. Lake water is denoted by black symbols, sediments are gray, and controls are white. Inset contains the 3.0, 0.8, and 0.2 μm samples from each water cast outlined with a black box.

The OTU composition and abundances in samples from the water column were significantly different from those of the sediment (*p* ≤ 0.005), with the surficial horizon (0–2 cm) showing the highest similarity to the lake water (**Figure 1**). Both the lake water and sediment communities were statistically distinct from those in the drill and borehole water (*p* ≤ 0.008), and grouped distinctly based on NMDS analysis (**Figure 1**). A detailed description of the microbial assemblages found in the drill system, borehole, and methodological controls are provided in the supplemental material (Supplemental Figure S1B). Overall, the SLW microbial community structure inferred from the analysis of rRNA and rDNA based data were not statistically different from one another (*p* = 0.12).

**FIGURE 2:**
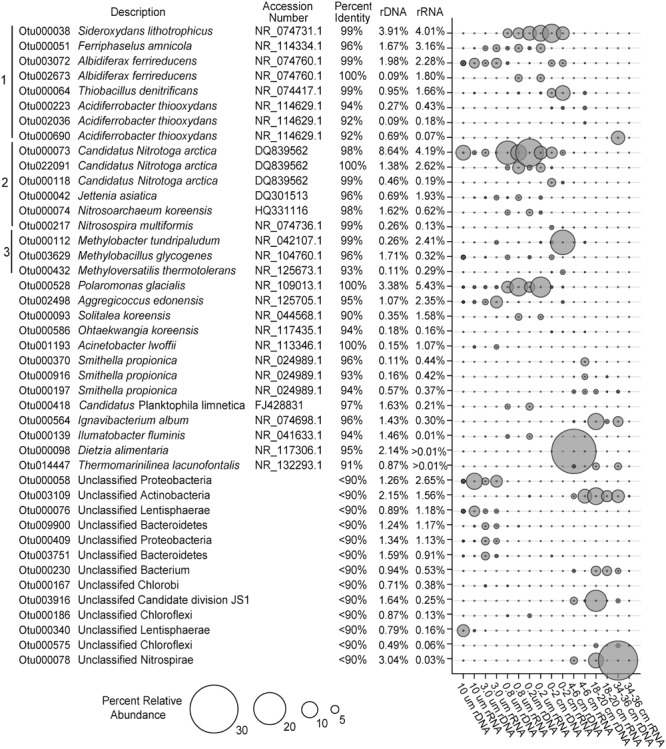
**The most abundant OTUs in the bulk SLW water column and sediments (>1%).** All of the OTUs are listed with their nearest taxonomic neighbor and total relative abundance in the overall SLW community. Brackets 1, 2, and 3 represent putative taxa that are involved in sulfur/iron, nitrogen, and methane cycling, respectively.

The vast majority of sequences obtained from the SLW water column and sediments could be taxonomically assigned at the domain level (<0.2% were unclassified). The SLW water column was dominated by bacteria, with only 3 and 2% of the OTUs classifying as Archaea in the rDNA and rRNA libraries, respectively, whereas <0.3% of the sediment community was archaeal. The phylum Thaumarchaeota comprised ∼98% of archaeal OTUs within the water column rRNA libraries, while Euryarchaeota represented 85% of archaeal sequences obtained from the sediments. The most abundant archaeal OTU within the SLW water column (OTU000074) comprised 1.6% of the rDNA and 0.6% of the rRNA-based sequences, respectively, and classified within the genus *Candidatus* Nitrosoarchaeum (**Figure 2**). The most abundant euryarchaeotal OTU in the rRNA libraries is most closely related to *Methanohalophilus levihalophilus* (91% sequence identity) and was only detected in the 34–36 cm sediment horizon.

Proteobacteria were the dominant phylum in the SLW ecosystem. Water and surficial sediments (0–2 cm) were primarily comprised of Betaproteobacteria, while the abundance of Gammaproteobacteria generally increased with sediment depth (Supplementary Figure S2). The most abundant OTU (OTU000528; ∼8% rRNA and ∼5% rDNA) in the water column community was 100% identical to *Polaromonas glacialis* (**Figure 2**). Many of the OTUs abundant in the lake were rare (<0.01%) in the sediment community, including taxa most closely related to species of *Ferriphaselus* (96% identity; OTU000051) and *Solitalea* (94%; OTU000093), as well as several poorly classified members of the Proteobacteria and Lentisphaerae (**Figure 2**). Also prevalent in the lake were several OTUs (OTUs 000073, 000118, and 022091) sharing ≥98% identity to *Candidatus* Nitrotoga arctica (**Figure 2**), representing 9 and 13% of the rRNA and rDNA water sequences, respectively. This taxa was also found in the sediment community (2% in rRNA and 5% in rDNA at 0–2 cm), but their abundance decreased rapidly with depth (**Figure 2**).

Phylotypes in the genera *Albidiferax, Sideroxydans, Thiobacillus*, and *Methylobacter* were shared between the water column and surficial sediments (0–2 cm). An OTU (000038) having 99% identity with *Sideroxydans lithotrophicus* was the third most abundant OTU in both the water column and 0–2 cm sediment horizon (rRNA; **Figure 2**). Two OTUs (002673 and 003072) closely related to *Albidiferax ferrireducens* (≥99% identity), were also highly prevalent in the water column (5% of rRNA and 3% rDNA sequences) and sediments (∼1% of the rRNA and DNA sequences). The *Albidiferax*-related OTUs also showed a preference based on size, with larger representation in the 10 and 3 μm rRNA libraries (**Figure 2**). *Methylobacter*- (OTU000112) and *Thiobacillus*-related (OTU000064) taxa were also present in the water column rRNA libraries (2 and 1%, respectively), but their abundance was ∼10-fold higher in the surficial sediments (0–2 cm; **Figure 2**).

At sediment depths between 4 and 36 cm, several abundant OTUs in the rRNA libraries (OTUs 000197, 000370, and 000916) most closely related to *Smithella propionica* (≥93% identity) comprised 4% of the sediment rRNA sequences (**Figure 2**). Also abundant in the deeper horizons (>6 cm) was an OTU (000564) 96% identical to *Ignavibacterium album* and one that classified as a member of the Candidate division JS1 (OTU003961; **Figure 2**). When compared to the water column, the Candidate division JS1, Nitrospirae, and Gammaproteobacteria were generally higher in abundance in the sediments (Supplementary Figure S2).

### 16S rRNA:rDNA Ratios

**FIGURE 3:**
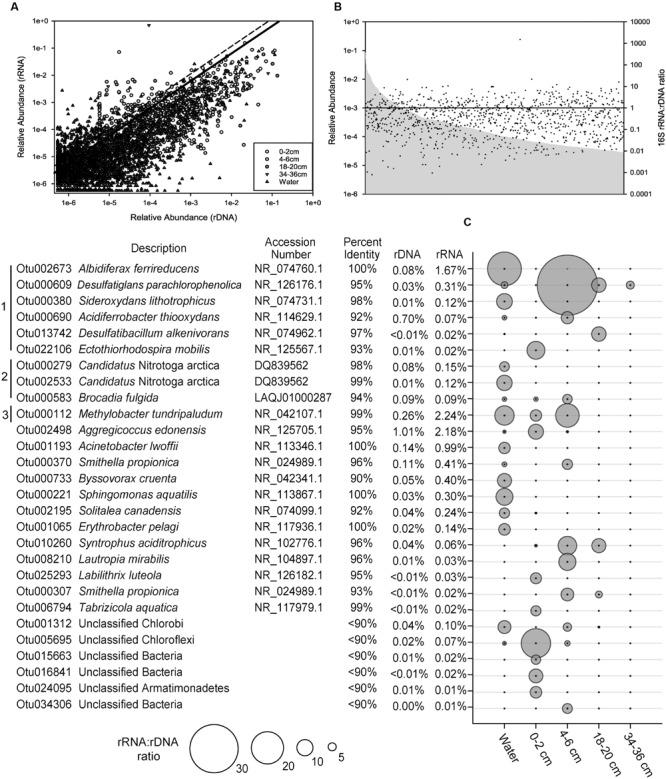
**The ratio of 16S rRNA to rDNA sequences obtained from the SLW molecular data. (A)** The relative abundance of individual OTUs in rRNA versus the rDNA sequence data. The black line denotes those OTUs in the top 1% (ratio of 5.8) while those above the hashed line represent outliers (ratio >10.7). **(B)** The rank abundance of OTUs (gray), based on 16S rDNA data, and their rRNA:rDNA ratios denoted as black dots. The horizontal black line denotes an rRNA:rDNA ratio of 1. **(C)** List of the top 1% of OTUs with rRNA:rDNA ratios ≥5.8, identified by their nearest taxonomic neighbor. Bracket designations are the same as **Figure 2**.

The ratio of 16S rRNA to rDNA sequence abundance for each OTU identified in SLW was examined. Of the OTUs detected in both the rRNA and rDNA based libraries, 59% had ratios greater than 1, and there was a positive correlation (Pearson Correlation; *r* = 0.77) between the abundance of rRNA and rDNA sequences for individual OTUs (**Figure 3A**). No correlation was observed between the relative abundance of OTUs and their rRNA:rDNA ratios (**Figure 3B**).

The relative abundance of 16S rRNA to rDNA sequences for a given OTU can be an indication of their potential for metabolic activity (Blazewicz et al., 2013); therefore, OTUs with the largest ratios (≥5.8; 99th percentile) were examined in greater detail. Several of the numerically abundant phylotypes (e.g., species of *Albidiferax, Methylobacter, Candidatus* Nitrotoga, *Sideroxydans*, and *Smithella*) were also in the top 1% of the community based on high rRNA:rDNA ratios (**Figure 3C**). Rarer taxa, such as those closely related to *Desulfatiglans parachlorophenolica* (95% identity; OTU000609) and a poorly classified member of the Chloroflexi (OTU005695), were among the OTUs with the largest rRNA:rDNA abundance ratios observed (38 and 18, respectively).

## Discussion

Antarctic subglacial lakes have been a scientific curiosity since their discovery (Oswald and Robin, 1973). The initial studies on accretion ice from Subglacial Lake Vostok concluded that viable microbes were present in the surface waters of the lake (Karl et al., 1999; Priscu et al., 1999), but due to concerns about sample contamination (e.g., Christner et al., 2005), evidence supporting the habitability of Antarctica’s subglacial environment was subject to criticism. More recently, studies have demonstrated the suitability of subglacial environments as ecosystems and shown that liquid water reservoirs at the base of glaciers harbor diverse assemblages of microorganisms (e.g., Skidmore et al., 2005; Boyd et al., 2011; Hamilton et al., 2013; Dieser et al., 2014). However, progress in understanding Antarctic subglacial lake microbiology has been hampered by logistical constraints that make sampling and direct observations challenging. The WISSARD project collected the first pristine water and sediment samples from an Antarctic subglacial lake (Christner et al., 2014), providing an unprecedented opportunity to examine microbial community structure and infer ecosystem processes in an aquatic habitat beneath the West Antarctic Ice Sheet (WAIS).

Conclusions on the composition of SLW microbial communities can be made with confidence given the significant differences they displayed from assemblages in the drilling water and procedural controls (**Figure 1**; Supplementary Results). With the exception of *Janthinobacterium, Tumebacillus*, and *Herbaspirillum*-related OTUs, OTUs identified in the drilling water were rare or absent in lake water samples. Microorganisms that were present in the borehole and drill water may be human-based contaminants, derived from the drill system plumbing, and/or have originated from the snow and glacial ice that sourced the water for the drill (Blythe et al., 2014; Rack et al., 2014). The latter is supported by the abundance of OTUs related to *Tumebacillus* and *Janthinobacterium* in drilling and borehole water, as these taxa have been frequently identified in polar environments (e.g., Steven et al., 2008; Kim et al., 2012). Alternatively, the presence of OTUs related to *Herbaspirillum*, a bacterial genus that frequently contaminates DNA extraction kits (Salter et al., 2014), may be an artifact of laboratory contamination. Determining the conclusive source of the *Herbaspirillum* phylotypes is complicated by the fact that limnological studies of surface Antarctic lakes have identified these taxa as bona fide members of the bacterioplankton communities (Pearce et al., 2003; Kuhn et al., 2014). Given these uncertainties, all of the OTUs discussed above were not considered to be members of the SLW community.

In the absence of light and photosynthetic activity, chemolithoautotrophic species may play a crucial role in carbon cycling through the generation of new organic carbon for the subglacial ecosystem. Christner et al. (2014) concluded that rates of CO_2_ fixation in samples from the SLW water column were sufficient to support heterotrophic production. Given that the waters of SLW are predominantly derived from glacial melt, the bulk of nutrients needed to fuel primary production and support the ecosystem are likely sourced from subglacial sediments that contain relict marine organic matter deposits which are widespread beneath this portion of the WAIS (Wadham et al., 2012; Christner et al., 2014). Hence, the underlying geology and historical connection to the Ross Sea was expected to strongly influence the structure and metabolic function of microorganisms inhabiting the SLW ecosystem through nutrient input.

Similar to other subglacial environments (Christner et al., 2001; Skidmore et al., 2005; Lanoil et al., 2009; Hamilton et al., 2013; Dieser et al., 2014), the microbial species of SLW were largely composed of Proteobacteria, Actinobacteria, Bacteroidetes, and Firmicutes (Supplementary Figure S2), and also contained members of the Nitrospirae, Chloroflexi, Chlorobi, Thaumarchaeota, and Candidate Division JS1 phyla (Supplementary Figure S2). The most abundant taxa in SLW (*Polaromonas, Sideroxydans*, and *Thiobacillus*) were closely related to sequences characterized from sediment cores recovered from beneath the neighboring Kamb Ice Stream (Lanoil et al., 2009), while *Methylobacter, Albidiferax, Candidatus* Nitrotoga, and Thaumarchaeota species have been shown to be prevalent in subglacial outflows, basal ice, permafrost, and polar waters (Alawi et al., 2007; Cheng and Foght, 2007; Alonso-Sáez et al., 2011; Dieser et al., 2014; Doyle, 2015). The widespread distribution of these taxa in cold environments suggests an inherent tolerance of conditions in the cryosphere, and that they may serve important ecological functions in the subglacial habitat.

**FIGURE 4:**
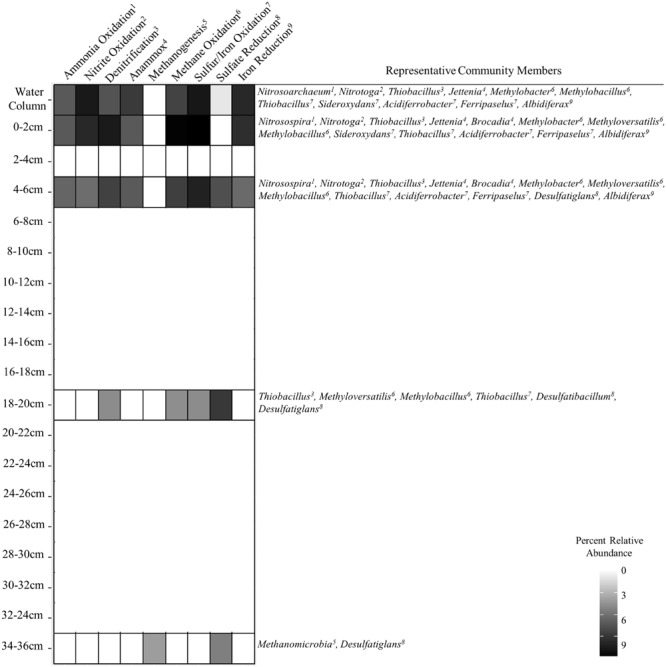
**Heat plot showing the occurrence of various putative metabolic functions in SLW inferred from 16S rRNA phylogenetic associations and sequence abundances of important community members**.

The relationship between abundances of sequenced 16S rRNA molecules and genes has been used as a means to assess the metabolic activity of individual taxa (e.g., DeAngelis et al., 2010; Jones and Lennon, 2010; Campbell et al., 2011; Dieser et al., 2014). Although cellular ribosome concentration correlates positively with growth rate, the interpretation of rRNA:rDNA ratios in natural communities is more complex because the relationship is not uniform for all species (Blazewicz et al., 2013) and may require a fundamentally different interpretation for oligotrophic versus copiotrophic lifestyles (Lankiewicz et al., 2015). In light of these uncertainties, high 16S rRNA abundance and rRNA:rDNA ratios were evaluated to specifically identify outliers in the SLW community data. The OTUs identified through this analysis are inferred to have the greatest potential for metabolic activity within the community and thus most likely to be influencing biogeochemical transformations in SLW at the time it was sampled. The positive correlation between the abundance of rRNA and rDNA in the SLW OTUs (**Figure 3A**) supported that taxa abundant in the rDNA data were ecologically important and unlikely to represent dead cell populations. Rank abundance of the taxa did not correlate with the rRNA:rDNA ratio (**Figure 3B**), suggesting that abundance per se was not a good predictor for potential metabolic activity. Although assumptions about metabolic function based on short 16S rRNA gene fragments are tenuous, many of the numerically abundant (**Figure 2**) and rare OTUs (**Figure 3C**) with large rRNA:rDNA ratios (>5.8; **Figure 3C**) were phylogenetically related to bacterial and archaeal species that use reduced nitrogen, iron, and sulfur compounds or C1 compounds as primary electron donors (**Figure 4**).

Geochemical analysis of the dissolved inorganic nitrogen pool in the SLW water column showed that it was comprised primarily of ammonium (∼2.4 μmol L^-1^) and nitrate (0.8 μmol L^-1^), with Δ^17^O-NO_3_^-^ values (-0.1 to 0.2‰) indicating *in situ* nitrification (Christner et al., 2014). The potential for nitrification was also supported by the abundance of taxa classifying within the Thaumarchaeota (Supplementary Figure S2; **Figure 3**), a ubiquitous clade containing chemolithotrophic (Könneke et al., 2005) and mixotrophic (Qin et al., 2014) archaea that derive energy from ammonia oxidation. Water column OTUs related to the ammonia oxidizing archaea (AOA) were predominately found as cell populations that passed through 3.0 μm pores and were primarily retained on the 0.8 and 0.2 μm filters (**Figure 2**), which agreed well with individual cell sizes reported for this group (<1 μm, Könneke et al., 2005). The AOA were 1.6- and 14-times (rRNA and rDNA, respectively) more abundant than known ammonia oxidizing bacterial (AOB) genera in the water column; however, the abundance of AOA quickly diminished with sediment depth, while the AOB increased (55-fold higher in 0–2 cm rRNA, **Figures 2** and **4**). The most abundant AOB-related OTU in the sediments had 99% sequence identity with *Nitrosospira multiformis* (**Figure 2**). AOA have been found to outcompete AOB in environments with low ammonium concentrations (<1 μM), presumably due to the higher affinity of their ammonia monooxygenase for substrate (Martens-Habbena et al., 2009). This fact may explain the distribution of AOA and AOB in SLW, as ammonium was ∼40-times more abundant in sediment pore waters than in the lake (Vick-Majors, 2016). Further, SLW contained abundant and rare OTUs with large rRNA:rDNA ratios that were related to the anaerobic ammonia oxidizing bacteria (**Figure 4**) *Candidatus* Jettenia asiatica (OTU000042; **Figure 2**; Hu et al., 2012) and *Brocadia fulgida* (OTU000583; **Figure 3C**; Kartal et al., 2008), respectively. These microorganisms may serve an important role in nitrogen removal within the sediments during conditions of water column hypoxia.

With only one known exception (i.e., van Kessel et al., 2015), the second step of nitrification is catalyzed by a physiologically distinct group of microorganisms that oxidize nitrite to nitrate. Based on the SLW water column data, we conclude that this reaction is probably carried out by a group of Betaproteobacteria closely related to *Candidatus* Nitrotoga arctica, a nitrite-oxidizing isolate from Siberian permafrost (Alawi et al., 2007; **Figure 4**). OTUs related to *Candidatus* Nitrotoga were highly abundant in the water column (∼10% rRNA) and surficial sediment (0–2 cm; 3.8% rRNA; **Figure 4**). Also prevalent within the surficial sediments (0–6 cm) were OTUs closely related to *Thiobacillus denitrificans* (99% sequence identity; **Figures 2** and **4**), a microorganism with the capacity to use inorganic sulfur compounds or Fe^2+^ as an electron donor via aerobic respiration or denitrification (Beller et al., 2006).

Studies of subglacial sediments from alpine glaciers (e.g., Skidmore et al., 2005; Boyd et al., 2014; Harrold et al., 2016) and the WAIS (Lanoil et al., 2009) have provided evidence that sulfide and iron oxidation are microbially mediated weathering processes that occur at the bed. Boyd et al. (2014) inferred microbial pyrite oxidation beneath the Robertson Glacier based on geochemical and functional gene analysis, concluding that it may contribute to subglacial primary production. Within SLW’s pyrite containing sediments (Michaud et al., 2016), species of *Sideroxydans, Ferriphaselus*, and *Albidiferax* (**Figures 2–4**; Kato et al., 2014) could participate in iron cycling. *A. ferrireducens* is a heterotrophic, facultative anaerobe capable of coupling the reduction of Fe^+3^ to the oxidation of organic compounds such as acetate (Finneran et al., 2003), which was ∼1 μM in the SLW water column (Christner et al., 2014; **Figure 4**). *Albidiferax*-related OTUs were found within the surficial sediments, but in the water column, the majority were observed on the 10 and 3.0 μm filter size fractions, consistent with the size reported for the type strain (3–5 μm filaments; Finneran et al., 2003) and/or attachment to water column suspensoids. Although iron reduction is thermodynamically unfavorable in the oxic water column, members of *Albidiferax* may be associated with anaerobic microhabitats within large suspended sediment particles as has been found in marine snow aggregates (e.g., Stief et al., 2016). It is notable that members of this genera have also been found abundant in other glacier ecosystems, including basal ice facies from the Matanuska Glacier, Alaska and periglacial streams near Thule, Greenland (Doyle, 2015).

Sequences related to the adenosine-5′-phosphosulfate reductase (*aprA*) gene, a key enzyme involved in sulfur redox chemistry, of *Sideroxydans* and *Thiobacillus* species were detected in SLW sediments (Purcell et al., 2014). Purcell et al. (2014) used quantitative PCR and estimated 0.084 (28–34 cm) to 9.2 × 10^5^ (0–4 cm) *aprA* gene copies per gram of wet SLW sediment, which represented from 0.9% (28–34 cm) to 15% (0–4 cm) of the 16S rRNA gene copies that were also detected. This is consistent with our rDNA analysis which showed that OTUs related to the sulfur oxidizing species *Sideroxydans* and *Thiobacillus* accounted for 18% of rDNA sequences in the 0–2 cm sediment horizon (17% rRNA), decreasing to 0.01% at a depth of 34–36 cm (0% rRNA; **Figure 2**). Members of these genera were also inferred to be highly abundant in the SLW water column (OTUs000038 and 000064; **Figure 2**) and had large rRNA:rDNA ratios (OTU000380; **Figures 3C** and **4**). The activity of sulfide oxidizing microorganisms within SLW sediments is further supported by the excesses of crustally derived sulfate observed in the upper 15 cm of the sediments (Michaud et al., 2016). Purcell et al. (2014) also measured low rates of sulfate reduction in laboratory experiments with SLW sediments. Although known sulfate reducing bacteria were rare in the SLW sediment community, OTUs related to the genera *Desulfatiglans* and *Desulfatibacillum* had high rRNA:rDNA ratios in the 4–6 and 16–18 cm sediment horizons, consistent with their potential to conduct sulfate reduction in the SLW sediments if redox conditions within the lake become favorable (**Figure 4**). Nonetheless, sulfate profiles in the sediment porewaters suggested sulfate reduction was not an active process in the upper 36 cm of the sediments (Michaud et al., 2016). Consequently, the *in situ* function of these organisms is unknown.

The basal sediments beneath ice sheets may harbor globally significant reservoirs of organic matter and be methane sources to the atmosphere (Wadham et al., 2008). Wadham et al. (2012) estimated a methane reservoir of ∼10 Pg C beneath the WAIS, where thick organic rich sediments combined with low temperature and high pressure would promote methane hydrate storage. Methanogenesis has been reported in a number of subglacial environments (Boyd et al., 2010; Stibal et al., 2012a,b), including the bed of the Greenland Ice Sheet (Christner et al., 2012; Dieser et al., 2014). In the sediment core analyzed for this study, OTUs related to methanogenic archaea were rare (0.1%) and only detected in the lowest sediment depth analyzed (34–36 cm). This coupled with measurements of methane in sediment pore water suggests that methanogenic populations and activity may have been more prevalent in deeper portions of SLW’s sediments (Michaud, 2016; **Figure 4**). An OTU (000112) closely related to the type I methanotroph, *Methylobacter tundripaludum* (99% identity), was ∼10-fold lower in the water column than in the 0–2 cm sediment rRNA where it represented ∼15% of the sequences (**Figure 2**). Dieser et al. (2014) identified a similar phylotype of *Methylobacter* that was inferred to be responsible for methane oxidation (320 nM CH_4_ d^-1^) in subglacial water outflows at the western margin of the Greenland Ice Sheet. The presence of active methanotrophy in SLW was supported not only by the high abundances of methanotroph-related OTUs and their large rRNA:rDNA ratios (**Figure 3C**), but also by geochemical and isotopic data which showed a significant decrease in methane concentration between the surficial sediments (0–2 cm) and the water column concurrent with a positive shift in the δ^13^C-CH^4^ (Michaud, 2016). Together, these results imply that microbial methane oxidation may serve a role as a substantial methane sink beneath the WAIS. The effect of subglacial methane release to the atmosphere during ice sheet wastage is estimated to be significant (e.g., Wadham et al., 2012), but such efforts have not considered aerobic methane oxidation as an aspect of carbon cycling beneath ice masses. Our data and those of Dieser et al. (2014) imply that bacterial methane consumption could be a significant methane sink and pathway for primary production in portions of the subglacial hydrological system where methane and oxygen coexist.

## Conclusion

There is a lack of fundamental information on the microbial biomes at the base of polar ice sheets because accessing these subglacial environments in a clean, environmentally conscious way is technically and logistically challenging. The WISSARD project at SLW provided the first opportunity to examine the limnology and ecology of an Antarctic subglacial lake, revealing a chemosynthesis-based community comprised of bacteria and archaea, with no conclusive evidence for the presence of eukaryotic species (Christner et al., 2014; Achberger, 2016). The members of SLW’s microbial community appear to derive their nitrogen, iron, sulfur, and carbon compounds from mineral weathering and relict organic matter in the sediments (**Figure 4**; Michaud et al., 2016), but various nutrients may be actively cycled in SLW and the interconnected WIS subglacial hydrologic system. At present it is unclear how representative the SLW microbial ecosystem is of other lakes in this region or to the many hundreds of others beneath the Antarctic ice sheet. However, taxa phylogenetically related to many of the dominant OTUs present in SLW have been previously observed in other icy subsurface environments, raising possibilities for microbial biogeographical studies of specific taxa endemic to the polar regions. Most species inhabiting the SLW ecosystem were distinct from those entrapped in the overlying glacial ice (i.e., the water melted for drilling), implying that microbial inocula for the lake is derived from another source. Hence, microorganisms may enter into SLW as plankton or attached to particles that are transported by subglacial water source from more internal regions of the Antarctica continent.

## Author Contributions

All authors contributed to the study design, acquisition of samples, and writing of the manuscript. The generation and analysis of molecular data was conducted by AA.

## Conflict of Interest Statement

The authors declare that the research was conducted in the absence of any commercial or financial relationships that could be construed as a potential conflict of interest.
