# Supplementary material for: Microbial Community Structure of Subglacial Lake Whillans, West Antarctica
Source: Front Microbiol. 2016 Sep 22;7:1457. doi: 10.3389/fmicb.2016.01457 (PMC5032586; doi:10.3389/fmicb.2016.01457)
Supplement: Supplementary file 1 [file Presentation_1.PDF]

Supplemental Material

Microbial Community Structure of Subglacial Lake Whillans, West Antarctica

Amanda M. Achberger, Brent C. Christner*, Alexander B. Michaud, John C. Priscu, Mark L. Skidmore, Trista J. Vick-Majors, and the WISSARD Science Team

***Correspondence:** Brent C. Christner: xner@ufl.edu

1 Supplementary Data

1.1 Results

1.1.1 SLW 16S rDNA and rRNA sequence Diversity and Comparison

After quality filtering removed 28% of the sequence reads, 7,520,040 sequences (65% and 35% of the total rDNA and rRNA sequences, respectively) remained for further analysis. Calculations of sequence coverage (Good's Coverage; Supplemental Table 1) and rarefaction analysis (data not shown) indicated that the data were sufficient to characterize the dominant microbial community members. The rDNA-derived libraries from the lake water had lower species richness compared to the rRNA based libraries based on the number of observed OTUs and Chao1 richness estimates (Supplemental Table 1). In general, each of the indices for the rDNA water samples implied that the microbial diversity decreased with filter pore size. This trend was not observed in the rRNA samples, the diversity of which was more uniform across the size fractions. Although sediment richness (observed OTUs and Chao1) was lower in the rRNA versus rDNA data, both richness and estimations of diversity (Inv. Simpson and Shannon) were higher in surficial sediments (0-6cm) than in the deeper horizons (18-36cm; Supplemental Table 1).

1.1.2 Characterization of microorganisms in the drilling water and procedural controls

Comparative analysis of sequences amplified from extracted nucleic acids in water samples that circulated in the drilling system and borehole, extraction blanks, and SLW water and sediments was conducted to assess exchange with the lake and examine the source of phylotypes encountered during scientific operations at SLW. Samples collected at two time points (designated T1 and T2) and locations in the WISSARD water treatment and drilling system (Supplemental Figure 1A) were used to characterize the composition of microorganisms circulating in the drill water, and an additional sample was collected via hydrocast from a depth of 672 mbs in the borehole prior to SLW entry. Betaproteobacteria were the dominant phyla detected in the drilling system (45-84% of the rDNA sequences; Supplemental Figure 1B), being most abundant in water samples obtained prior to filtration and UV treatment (port 1, T1P1; Supplemental Figure 1A), and lowest in the borehole hydrocast sample (Supplemental Figure 1B). The most abundant betaproteobacterial OTUs in the drilling water were most closely

related to species of *Janthinobacterium* (23% of sequence reads), *Delftia* (12%), and *Herbaspirillium* (6%). Members of the *Janthinobacterium* and *Herbaspirillium* were also among the dominant betaproteobacterial OTUs in the borehole hydrocast sample (2% and 5%, respectively). Although the relative abundance of Alpha- and Gammaproteobacteria in the borehole sample was 16% and 17%, respectively, these classes were found to be consistently lower in the other drilling water samples (5 to 10%, respectively).

OTUs classifying within the Bacteriodetes and Firmicutes were highly abundant in the borehole hydrocast (12 and 39%, respectively) and return well samples (T1P9; 15 and 19% respectively), but were rare in samples from port 1 (Supplemental Figure 1B; 0.7 and 0.06%, respectively). During the second collection time point, the relative abundance of Bacteriodetes phylotypes decreased by ~7-fold in water returning to the surface via the return well pump (T2P9; Supplemental Figure 1B) compared to initial sampling (T1P9). A large portion of the Firmicutes sequences (19%) that were associated with the drilling and borehole water grouped within a single OTU affiliated with the genus *Tumebacillus,* while *Cloacibacterium* and *Chyseobacterium*-like taxa were the most abundant Bacteriodetes (3.34% and 3%, respectively, of all drill water sequences). OTUs within the genera *Tumebacillus* and *Chryseobacterium,* along with those closely related to *Janthinobacterium* and *Delftia,* were also found in all the lake water samples, and a *Herbasparillium*-like OTU was observed in every rDNA derived library. Combined, these 5 OTUs represented between 37 and 78% of the sequences obtained from the drilling water samples, and between <1% and 27% of the sequence reads from lake water and sediment samples (average of 3%). Despite the presence of several shared OTUs, microbial assemblages in the drilling water were statistically different from those of the lake water and sediment ($p \leq 0.008$), and grouped distinctly in a nonmetric multidimensional scaling (NMDS) analysis (Figure 1).

Procedural blanks were processed using materials and solutions that were identical to those used for extractions of SLW lake water and sediment samples. No PCR amplification was observed in any DNA-based controls that targeted 16S rRNA genes (Christner et al, 2014), and therefore, none were available for sequencing. Weak amplification was observed from blanks processed for the extraction and detection of 16S rRNA molecules, and four of these samples were selected for sequencing and analysis. The blank controls shared 2% of the water column OTUs from the rRNA based libraries and 14% of those from the sediments. Less than 3% of the OTUs in the blanks were shared with those in rDNA derived libraries and were considered likely contaminants that originated from procedures associated with nucleic acid extraction, amplification, and/or sequencing. Hence, they were not considered further in the SLW microbial community description. The majority of OTUs from the rRNA controls classified as Gammaproteobacteria (average of 57%), while 21% was comprised of Betaproteobacteria, and 11% of Firmicutes. A large portion of the Gammaproteobacterial reads classified within three OTUs closely related to species of *Halomonas* (15% of the rRNA control reads), *Escherichia* (10%), and *Pseudomonas* (5%). The other abundant OTUs in the control samples were related to an unclassified member of the order *Vibrionales* (8%) and a *Herbaspirillium* species (9%).

References

Caporaso JG, Lauber CL, Walters WA, Berg-Lyons D, Huntley J, Fierer N *et al* (2012). Ultra-high-throughput microbial community analysis on the Illumina HiSeq and MiSeq platforms. *The ISME journal* 6: 1621-1624.

Christner BC, Priscu JC, Achberger AM, Barbante C, Carter SP, Christianson K *et al* (2014). A microbial ecosystem beneath the West Antarctic ice sheet. *Nature* 512: 310-313.

Dieser M, Broemsen EL, Cameron KA, King GM, Achberger A, Choquette K *et al* (2014). Molecular and biogeochemical evidence for methane cycling beneath the western margin of the Greenland Ice Sheet. *The ISME journal* 8: 2305-2316.

Priscu JC, Achberger AM, Cahoon JE, Christner BC, Edwards RL, Jones WL *et al* (2013). A microbiologically clean strategy for access to the Whillans Ice Stream subglacial environment. *Antarct Sci* 25: 637-647.

Rack FR (2016). Enabling clean access into Subglacial Lake Whillans: development and use of the WISSARD hot water drill system. *Phil Trans R Soc A* 374: 20140305.

2 Supplementary Figures and Tables

2.1 Supplementary Tables

Supplemental Table 1. Summary of parameters for 16S rRNA and rRNA gene analysis for SLW separated according to cast, filter size fraction, and sediment depth. Indices were calculated on subsampled data within Mothur and are an average of 1000 iterations. Singletons were included in this analysis.

	**Observed OTUs**		**Inverse Simpson**		**Shannon Diversity**		**Chao1**		**Good's Coverage**	
	rDNA	rRNA	rDNA	rRNA	rDNA	rRNA	rDNA	rRNA	rDNA	rRNA
**0-2 cm**	98	449	4.8	17.9	2	3.9	185	630	99.6%	98.8%
**2-4 cm**	256	113	14.1	5.9	3.2	2.2	476	274	98.9%	99.4%
**4-6 cm**	69	375	1.6	20.8	1	3.8	119	518	99.7%	99.0%
**18-20 cm**	118	203	4.7	3.7	1.9	2.4	236	423	99.4%	99.2%
**34-36 cm**	133	144	3.7	1.8	2	1.3	183	302	99.6%	99.3%
**Cast 1 10.0 µm**	168	462	9.2	22	2.9	3.9	263	755	99.5%	98.3%
**Cast 1 3.0 µm**	36	68	4.5	7	1.9	2.3	45	125	99.9%	99.8%
**Cast 1 0.2 µm**	38	46	2.3	2.3	1.2	1.3	61	75	99.9%	99.8%
**Cast 2 3.0 µm**	264	306	14.7	9.6	3.2	2.8	418	883	99.0%	98.2%
**Cast 2 0.8 µm**	229	619	7.6	14.8	2.7	3.5	426	1574	99.0%	96.6%
**Cast 2 0.2 µm**	80	960	3.4	18.7	1.7	4	156	2754	99.7%	94.3%
**Cast 3 3.0 µm**	197	425	10.2	9.5	2.8	3	379	1157	99.1%	97.6%
**Cast 3 0.8 µm**	130	285	5	6.5	2.2	2.5	275	866	99.4%	98.4%
**Cast 3 0.2 µm**	61	791	2.5	11.7	1.4	3.6	104	2153	99.8%	95.4%

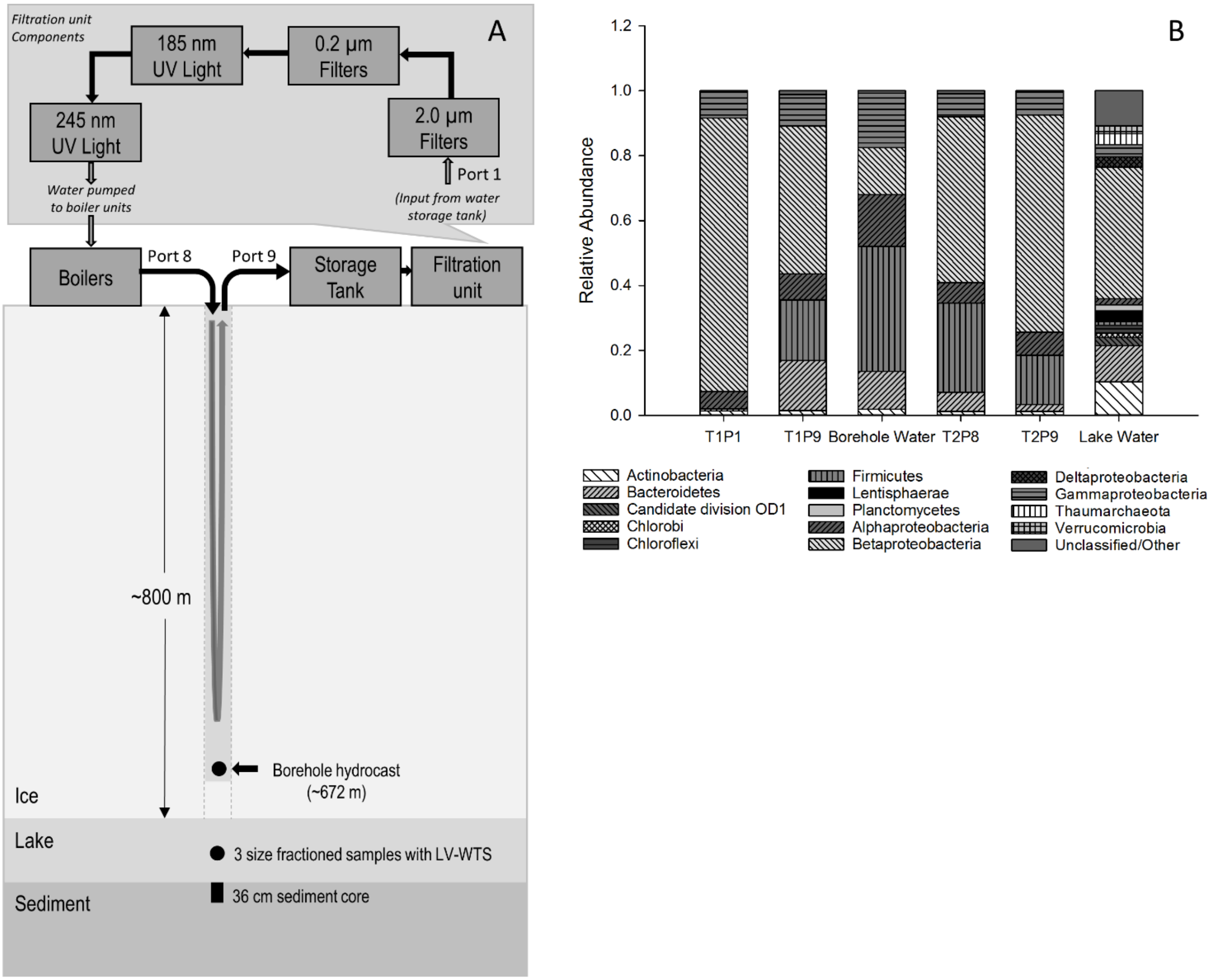

Supplemental Figure 1. The microorganisms associated with the hot water drill system. (A) Schematic representation of WISSARD's microbiologically-clean hot water drill system, showing the filtration and UV module components, location of sampling ports used, and the SLW access borehole. (B) The relative abundance of the dominant phylum (>1% of sequence reads) in drill water, borehole, and bulk water samples.

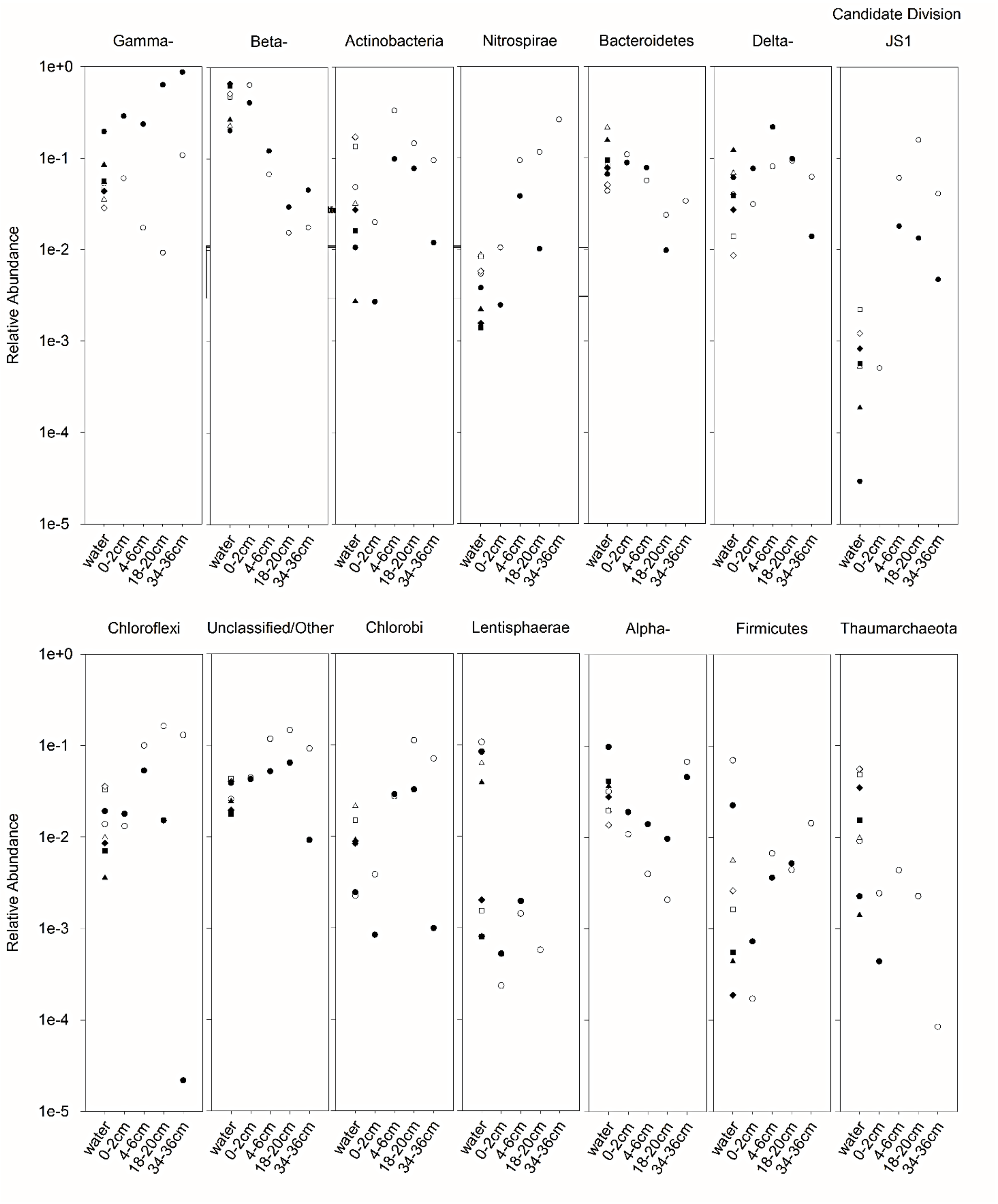

Supplemental Figure 2. The relative abundance of the 14 most abundant phyla in the SLW water column and sediments. Data from size fractions in the water column and sediment depths are indicated. White and black symbols denote 16S rDNA and rRNA sequence abundances,

respectively. Within the water the 10um size fraction is denoted by circles, the 3um by triangle, the 0.8um by square, and the 0.2um by diamond.
